# The Emerging Role of Histone Demethylases in Renal Cell Carcinoma

**DOI:** 10.15586/jkcvhl.2017.56

**Published:** 2017-05-03

**Authors:** Xiaoqiang Guo, Qiaoxia Zhang

**Affiliations:** 1 State Engineering Laboratory of Medical Key Technologies Application of Synthetic Biology, Key Laboratory of Medical Reprogramming Technology, Shenzhen Second People’s Hospital, The First Affiliated Hospital of Shenzhen University, Shenzhen, Guangdong, China; 2 Department of Urology, Shenzhen Second People’s Hospital, The First Affiliated Hospital of Shenzhen University, Shenzhen, Guangdong, China; 3 Laboratory of Molecular Iron Metabolism, College of Life Science, Hebei Normal University, Shijiazhuang, Hebei, China

**Keywords:** histone demethylases, KDM3A, KDM5C, KDM6A, KDM6B, renal cell carcinoma

## Abstract

Renal cell carcinoma (RCC), the most common kidney cancer, is responsible for more than 100,000 deaths per year worldwide. The molecular mechanism of RCC is poorly understood. Many studies have indicated that epigenetic changes such as DNA methylation, noncoding RNAs, and histone modifications are central to the pathogenesis of cancer. Histone demethylases (KDMs) play a central role in histone modifications. There is emerging evidence that KDMs such as KDM3A, KDM5C, KDM6A, and KDM6B play important roles in RCC. The available literature suggests that KDMs could promote RCC development and progression *via* hypoxia-mediated angiogenesis pathways. Small-molecule inhibitors of KDMs are being developed and used in preclinical studies; however, their clinical relevance is yet to be established. In this mini review, we summarize our current knowledge on the putative role of histone demethylases in RCC.

## Introduction

Renal cell carcinoma (RCC) accounts for 2%–3% of all adult malignancies and causes more than 100,000 deaths per year worldwide (1). Radical or partial nephrectomy of the tumor at an early stage remains the mainstay of curative therapy (2). Metastases are present at the time of initial diagnosis in approximately one-third of patients, which are generally resistant to chemotherapy and radiation therapy (3). A better understanding of molecular mechanisms of RCC is necessary for improvement of treatment outcomes. Epigenetics refers to functionally relevant changes in the genome that affect the expression of specific genes without the involvement of changes in the DNA sequence (4). The common epigenetic events include DNA methylation, changes in noncoding RNAs and posttranslational modifications (PTMs) of histone. PTMs of histone involve the covalent modification of histone through acetylation, methylation, and phosphorylation (5). PTMs of histone regulate DNA replication, transcription, and repair of many biological processes (6). Histone methylation, a process by which methyl groups are transferred to lysine or arginine residues of histone, is a type of PTM of histone. Histone methylation influences many biological processes in the context of development and cellular responses (7). Abnormality of histone methylation can lead to various disorders including cancer (8). In this mini review, we summarize the emerging role of histone demethylases, key players in histone methylation, in RCC.

## Histone Demethylases and RCC

In 2004, the first histone demethylase KDM1 was discovered, and in the earlier days histone methylation was thought to be irreversible (9, 10). In 2006, several jumonji C (JmjC)-domain-containing demethylases were identified (11, 12), and subsequent studies showed that histone methylation is reversible. Histone demethylases play a key role in eukaryotic transcription (activation or repression) and other chromatin-dependent processes such as chromosome condensation and DNA damage (13). These demethylases have been implicated in the control of gene expression and cell fate decisions (14, 15). Many histone demethylases have been linked to human diseases (15–17), including RCC (Table 1).

**Table 1:** Histone demethylases implicated in RCC.

Nomenclature	Official symbol	Substrate	Function	References
KDM3A	JMJD1A	H3K9me1/2	Metabolism, reproduction	(18, 19)
KDM5C	JARID1C	H3K4me1/2	Neural development	(20, 21)
KDM6A	UTX	H3K27me2/3	Development	(22)
KDM6B	JMJD3	H3K27me2/3	Inflammatory response, senescence	(22)

RCC, renal cell carcinoma.

### KDM3A

KDM3A (also named as JMJD1A, JHDM2A) is an H3K9me1/2 demethylase of JmjC family and plays an essential role in spermatogenesis and adipogenesis (13, 14). KDM3A is also involved in other cellular processes such as cell cycle, embryonic and adult stem cell renewal, and differentiation of vascular smooth muscle (15). KDM3A has been implicated in the development and progression of several malignancies, including hepatocellular carcinoma and gastric cancer (16, 17). We and other researchers have reported that overexpression of KDM3A is associated with RCC development (18, 19). RCC samples from patients showed a higher expression of KDM3A when compared with normal noncancerous regions of the kidneys. Furthermore, KDM3A was highly expressed around blood vessels of RCC samples (18). KDM3A was also associated with an increase in hypoxia-inducible factor 1-alpha (HIF-1α) (18). *In vitro* experiments with the RCC cell line 786-0 showed that KDM3A was higher in hypoxic conditions than in normoxic conditions. Taken together, these findings (18) suggest the potential role of KDM3A in RCC development and progression *via* hypoxia-mediated angiogenesis pathway.

### KDM5C

KDM5C (also known as JARID1C) is an H3K4me1/2 demethylase that plays an important role in brain development and function. Mutations of KDMC5C can lead to X-linked mental retardation (23). KDM5C abnormality was also associated with cancer development. For example, KDM5C was significantly upregulated in breast cancer tissues compared with paired normal breast tissues, and was positively correlated with metastasis (24). Inactivating mutations of KDM5C were identified in 101 clear cell RCC (ccRCC) cases using massive parallel sequencing technologies (20). Further studies in 132 ccRCC patients showed that KDM5C was mutated in 4% of the cases (21).

### KDM6A and KDM6B

KDM6A (also named as UTX) is an H3K27me2/3 demethylase, that is, essential for normal embryonic development and tissue-specific differentiation (25). Inactivating somatic mutations of KDM6A have been identified in RCC (26). Our results showed that expression of KDM6A is upregulated in RCC (22). KDM6B, also known as JMJD3, is another H3K27me2/3 demethylase that plays important roles in inflammatory response and senescence (27). We found that KDM6B is also overexpressed in RCC, and maybe involved in oncogene-induced senescence (22). Thus, both KDM6A and KDM6B appear to have a proto-oncogenic role in RCC.

## Possible Mechanisms of Histone Demethylases in RCC Development

RCC is a hypoxia-related cancer because inactivating mutations of the tumor suppressor von Hippel-Lindau (VHL) gene are frequent in RCC than in other cancers. VHL is a ubiquitin ligase and its inactivation leads to increased protein stability of HIF1-α (28). HIF can change global patterns of histone modifications through transactivation of several histone demethylases (29, 30). Histone demethylases such as KDM3A, KDM3B, KDM4B, KDM5A, and KDM6B have been identified as HIF regulated demethylases (31). KDM3A has been established as a hypoxia-induced demethylase by several researchers (32–35). Upregulation of KDM3A mRNA and protein could be observed in RCC cell lines (786-0) exposed to hypoxia (1% O2) or iron scavengers (deferoxamine treatment). There is a hypoxia response element in the promoter region of the KDM3A gene, which can be bound by HIF-1 (33, 35). KDM6B was recently identified as a new hypoxia-inducible histone demethylase (36, 37). The expressions of KDM6A and KDM6B are also regulated by nicotine and nickel (38, 39), which are thought to induce RCC (40).

Histone demethylases can act as coactivators of certain nuclear factors including androgen receptor (AR), estrogen receptor, and HIF-1α. KDM3A is not only the coactivator of AR (13) but also the coactivator of HIF-1α (41). KDM3A can further increase specific genes expression, such as GLUT3, adrenomedullin, c-Myc, FGF2, HGF, and ANG2 (41–43). VHL inactivation in RCC can decrease H3K4me3 levels through KDM5C, which alters gene expression including IGFBP3 and GDF15 (44). In contrast, KDM5C inactivation can lead to genomic instability in RCC (45). These findings indicate that several histone demethylases can be induced under hypoxia which in turn regulate the expression of cancer-related genes, and trigger RCC development.

## Is There a Therapeutic Potential for KDM Inhibitors in RCC?

Current targeted therapies for metastatic RCC mainly include mTOR inhibitors, VEGFA receptor tyrosine kinase inhibitors, and anti-VEGFA antibodies (46). However, their efficacies are limited, and there is a need to identify new targets. Histone demethylases are one of the promising targets (47). There is increasing interest in targeting KDMs with small molecules for therapeutic purposes (48). Several high-throughput screening strategies have been developed to screen for small-molecule inhibitors of KDMs (49). Many histone demethylase inhibitors are being developed and tested (50, 51), including GSK-J1/GSK-J4 (KDM6B inhibitor) and NSC 636819 (KDM4A/KDM4B inhibitor). Research has indicated that GSK-J4 has potent antitumor role both in cell lines and animal models of glioma by inhibiting the KDM6B activity and increasing H3K27 methylation (51). Although histone demethylase inhibitors have substantial medicinal potential for the treatment of cancer (52), the major challenge is that these inhibitors are either characterized by low specificity or that their target enzymes have low substrate specificity (53). Furthermore, to date, no conclusive data are available on the efficacy of histone demethylase inhibitors on RCC. However, given that several compounds such as vitamins C and D have regulatory effects on expression or activity of histone demethylases including KDM3A and KDM6B (54, 55), targeting histone demethylases appears to be a potential therapeutic option for RCC (56).

## Conclusion

The past decade has witnessed tremendous improvements in the management of metastatic RCC through the introduction of many targeted therapies in clinical practice. Despite this, metastatic RCC still remains a difficult disease to treat. Better understanding of the molecular mechanisms that govern RCC development and progression will enable the development of novel compounds. There is emerging evidence that histone demethylases play a role in the development and progression of RCC, at least in part, *via* hypoxia-mediated angiogenesis pathway. This is particularly important given that hypoxia-induced angiogenesis pathway plays a crucial role in RCC progression, largely mediated by aberrations of the VHL gene. Furthermore, most of the targeted therapies inhibit the angiogenesis pathway. Thus, inhibition of histone demethylases, because of their perceived role in hypoxia-mediated angiogenesis, is a promising field for further exploration. In addition, the role of currently available targeted therapies on histone demethylases is another area for future research.

## Conflict of interest

The authors declare no potential conflicts of interest with respect to research, authorship, and/or publication of this article.

## References

[1] Siegel RL, Miller KD, Jemal A Cancer statistics, 2015. CA Cancer J Clin. 2015;65(1):5–29. http://dx.doi.org/10.3322/caac.21254 25559415 10.3322/caac.21254

[2] Ficarra V, Schips L, Guille F, Li G, De La Taille A, Prayer Galetti T, et al. Multiinstitutional European validation of the 2002 TNM staging system in conventional and papillary localized renal cell carcinoma. Cancer. 2005;104(5):968–74. http://dx.doi.org/10.1002/cncr.21254 16007683 10.1002/cncr.21254

[3] Mohammed A, Shergill I, Little B Management of metastatic renal cell carcinoma: Current trends. Expert Rev Mol Diagn. 2009;9(1):75–83. http://dx.doi.org/10.1586/14737159.9.1.75 19099350 10.1586/14737159.9.1.75

[4] Jones PA, Baylin SB The fundamental role of epigenetic events in cancer. Nat Rev Genet. 2002;3(6):415–28. http://dx.doi.org/10.1038/nrg816 12042769 10.1038/nrg816

[5] Tessarz P, Kouzarides T Histone core modifications regulating nucleosome structure and dynamics. Nat Rev Mol Cell Biol. 2014;15(11):703–8. http://dx.doi.org/10.1038/nrm3890 25315270 10.1038/nrm3890

[6] Bannister AJ, Kouzarides T Regulation of chromatin by histone modifications. Cell Res. 2011;21(3):381–95. http://dx.doi.org/10.1038/cr.2011.22 21321607 10.1038/cr.2011.22 PMC3193420

[7] Greer EL, Shi Y Histone methylation: A dynamic mark in health, disease and inheritance. Nat Rev Genet. 2012;13(5):343–57. http://dx.doi.org/10.1038/nrg3173 22473383 10.1038/nrg3173 PMC4073795

[8] Black JC, Van Rechem C, Whetstine JR Histone lysine methylation dynamics: Establishment, regulation, and biological impact. Mol Cell. 2012;48(4):491–507. http://dx.doi.org/10.1016/j.molcel.2012.11.006 23200123 10.1016/j.molcel.2012.11.006 PMC3861058

[9] Pedersen MT, Helin K Histone demethylases in development and disease. Trends Cell Biol. 2010;20(11):662–71. http://dx.doi.org/10.1016/j.tcb.2010.08.011 20863703 10.1016/j.tcb.2010.08.011

[10] Shi Y, Lan F, Matson C, Mulligan P, Whetstine JR, Cole PA, et al Histone demethylation mediated by the nuclear amine oxidase homolog LSD1. Cell. 2004;119(7):941–53. http://dx.doi.org/10.1016/j.cell.2004.12.012 15620353 10.1016/j.cell.2004.12.012

[11] Tsukada Y, Fang J, Erdjument-Bromage H, Warren ME, Borchers CH, Tempst P, et al Histone demethylation by a family of JmjC domain-containing proteins. Nature. 2006;439(7078):811–16. http://dx.doi.org/10.1038/nature04433 16362057 10.1038/nature04433

[12] Dimitrova E, Turberfield AH, Klose RJ Histone demethylases in chromatin biology and beyond. EMBO Rep. 2015;16(12):1620–39. http://dx.doi.org/10.15252/embr.201541113 26564907 10.15252/embr.201541113 PMC4687429

[13] Okada Y, Scott G, Ray MK, Mishina Y, Zhang Y Histone demethylase JHDM2A is critical for Tnp1 and Prm1 transcription and spermatogenesis. Nature. 2007;450(7166):119–23. http://dx.doi.org/10.1038/nature06236 17943087 10.1038/nature06236

[14] Tateishi K, Okada Y, Kallin EM, Zhang Y Role of Jhdm2a in regulating metabolic gene expression and obesity resistance. Nature. 2009;458(7239):757–61. http://dx.doi.org/10.1038/nature07777 19194461 10.1038/nature07777 PMC4085783

[15] Kasioulis I, Syred HM, Tate P, Finch A, Shaw J, Seawright A, et al Kdm3a lysine demethylase is an Hsp90 client required for cytoskeletal rearrangements during spermatogenesis. Mol Biol Cell. 2014;25(8):1216–33. http://dx.doi.org/10.1091/mbc.E13-08-0471 24554764 10.1091/mbc.E13-08-0471 PMC3982988

[16] Yamada D, Kobayashi S, Yamamoto H, Tomimaru Y, Noda T, Uemura M, et al Role of the hypoxia-related gene, JMJD1A, in hepatocellular carcinoma: Clinical impact on recurrence after hepaticresection. Ann Surg Oncol. 2012;19 Suppl 3:S355–64. http://dx.doi.org/10.1245/s10434-011-1797-x 21607773 10.1245/s10434-011-1797-x

[17] Yang H, Liu Z, Yuan C, Zhao Y, Wang L, Hu J, et al Elevated JMJD1A is a novel predictor for prognosis and a potential therapeutic target for gastric cancer. Int J Clin Exp Pathol. 2015;8(9):11092–9. 26617828 PMC4637643

[18] Guo X, Shi M, Sun L, Wang Y, Gui Y, Cai Z, et al The expression of histone demethylase JMJD1A in renal cell carcinoma. Neoplasma. 2011;58(2):153–7. http://dx.doi.org/10.4149/neo_2011_02_153 21275466 10.4149/neo_2011_02_153

[19] Krieg AJ, Rankin EB, Chan D, Razorenova O, Fernandez S, Giaccia AJ Regulation of the histone demethylase JMJD1A by hypoxia-inducible factor 1 alpha enhances hypoxic gene expression and tumor growth. Mol Cell Biol. 2010;30(1):344–53. http://dx.doi.org/10.1128/MCB.00444-09 19858293 10.1128/MCB.00444-09 PMC2798291

[20] Dalgliesh GL, Furge K, Greenman C, Chen L, Bignell G, Butler A, et al Systematic sequencing of renal carcinoma reveals inactivation of histone modifying genes. Nature. 2010;463(7279):360–3. http://dx.doi.org/10.1038/nature08672 20054297 10.1038/nature08672 PMC2820242

[21] Gossage L, Murtaza M, Slatter AF, Lichtenstein CP, Warren A, Haynes B, et al Clinical and pathological impact of VHL, PBRM1, BAP1, SETD2, KDM6A, and JARID1c in clear cell renal cell carcinoma. Genes Chromosomes Cancer. 2014;53(1):38–51. http://dx.doi.org/10.1002/gcc.22116 24166983 10.1002/gcc.22116

[22] Shen Y, Guo X, Wang Y, Qiu W, Chang Y, Zhang A, et al Expression and significance of histone H3K27 demethylases in renal cell carcinoma. BMC Cancer. 2012;12:470 http://dx.doi.org/10.1186/1471-2407-12-470 23057811 10.1186/1471-2407-12-470 PMC3520868

[23] Kramer JM, van Bokhoven H Genetic and epigenetic defects in mental retardation. Int J Biochem Cell Biol. 2009;41(1):96–107. http://dx.doi.org/10.1016/j.biocel.2008.08.009 18765296 10.1016/j.biocel.2008.08.009

[24] Wang Q, Wei J, Su P, Gao P Histone demethylase JARID1C promotes breast cancer metastasis cells *via* down regulating BRMS1 expression. Biochem Biophys Res Commun. 2015;464(2):659–66. http://dx.doi.org/10.1016/j.bbrc.2015.07.049 10.1016/j.bbrc.2015.07.049 26182878

[25] Van der Meulen J, Speleman F, Van Vlierberghe P The H3K27me3 demethylase UTX in normal development and disease. Epigenetics. 2014;9(5):658–68. http://dx.doi.org/10.4161/epi.28298 24561908 10.4161/epi.28298 PMC4063824

[26] van Haaften G, Dalgliesh GL, Davies H, Chen L, Bignell G, Greenman C, et al Somatic mutations of the histone H3K27 demethylase gene UTX in human cancer. Nat Genet. 2009;41(5):521–3. http://dx.doi.org/10.1038/ng.349 19330029 10.1038/ng.349 PMC2873835

[27] Swigut T, Wysocka J H3K27 demethylases, at long last. Cell. 2007;131(1):29–32. http://dx.doi.org/10.1016/j.cell.2007.09.026 17923085 10.1016/j.cell.2007.09.026

[28] Baldewijns MM, van Vlodrop IJ, Vermeulen PB, Soetekouw PM, van Engeland M, de Bruïne AP VHL and HIF signalling in renal cell carcinogenesis. J Pathol. 2010;221(2):125–38. http://dx.doi.org/10.1002/path.2689 20225241 10.1002/path.2689

[29] Hancock RL, Dunne K, Walport LJ, Flashman E, Kawamura A Epigenetic regulation by histone demethylases in hypoxia. Epigenomics. 2015;7(5):791–811. http://dx.doi.org/10.2217/epi.15.24 25832587 10.2217/epi.15.24

[30] Xia X, Lemieux ME, Li W, Carroll JS, Brown M, Liu XS, et al Integrative analysis of HIF binding and transactivation reveals its role in maintaining histone methylation homeostasis. Proc Natl Acad Sci USA. 2009;106(11):4260–5. http://dx.doi.org/10.1073/pnas.0810067106 19255431 10.1073/pnas.0810067106 PMC2657396

[31] Melvin A, Rocha S Chromatin as an oxygen sensor and active player in the hypoxia response. Cell Signal. 2012;24(1):35–43. http://dx.doi.org/10.1016/j.cellsig.2011.08.019 21924352 10.1016/j.cellsig.2011.08.019 PMC3476533

[32] Beyer S, Kristensen MM, Jensen KS, Johansen JV, Staller P The histone demethylases JMJD1A and JMJD2B are transcriptional targets of hypoxia-inducible factor HIF. J Biol Chem. 2008;283(52):36542–52. http://dx.doi.org/10.1074/jbc.M804578200 18984585 10.1074/jbc.M804578200 PMC2662309

[33] Wellmann S, Bettkober M, Zelmer A, Seeger K, Faigle M, Eltzschig HK, et al Hypoxia upregulates the histone demethylase JMJD1A *via* HIF-1. Biochem Biophys Res Commun. 2008;372(4):892–7. http://dx.doi.org/10.1016/j.bbrc.2008.05.150 18538129 10.1016/j.bbrc.2008.05.150

[34] Sar A, Ponjevic D, Nguyen M, Box AH, Demetrick DJ Identification and characterization of demethylase JMJD1A as a gene upregulated in the human cellular response to hypoxia. Cell Tissue Res. 2009;337(2):223–34. http://dx.doi.org/10.1007/s00441-009-0805-y 19471969 10.1007/s00441-009-0805-y

[35] Pollard PJ, Loenarz C, Mole DR, McDonough MA, Gleadle JM, Schofield CJ, et al Regulation of Jumonji-domain-containing histone demethylases by hypoxia-inducible factor (HIF)-1alpha. Biochem J. 2008;416(3):387–94. http://dx.doi.org/10.1042/BJ20081238 18713068 10.1042/BJ20081238

[36] Lee HY, Choi K, Oh H, Park YK, Park H HIF-1-dependent induction of Jumonji domain-containing protein (JMJD) 3 under hypoxic conditions. Mol Cells. 2014;37(1):43–50. http://dx.doi.org/10.14348/molcells.2014.2250 24552709 10.14348/molcells.2014.2250 PMC3907005

[37] Guo X, Tian Z, Wang X, Pan S, Huang W, Shen Y, et al Regulation of histone demethylase KDM6B by hypoxia-inducible factor-2α. Acta Biochim Biophys Sin (Shanghai). 2015;47(2):106–13. http://dx.doi.org/10.1093/abbs/gmu122 25520177 10.1093/abbs/gmu122

[38] Guo X, Li X, Wang Y, Tian Z, Duan X, Cai Z Nicotine induces alteration of H3K27 demethylase UTX in kidney cancer cell. Hum Exp Toxicol. 2014;33(3):264–9. http://dx.doi.org/10.1177/0960327113499043 23925944 10.1177/0960327113499043

[39] Guo X, Zhang Y, Zhang Q, Fa P, Gui Y, Gao G, et al The regulatory role of nickel on H3K27 demethylase JMJD3 in kidney cancer cells. Toxicol Ind Health. 2016;32(7):1286–92. http://dx.doi.org/10.1177/0748233714552687 25427687 10.1177/0748233714552687

[40] Lipworth L, Tarone RE, McLaughlin JK The epidemiology of renal cell carcinoma. J Urol. 2006;176(6 Pt 1):2353–8. http://dx.doi.org/10.1016/j.juro.2006.07.130 17085101 10.1016/j.juro.2006.07.130

[41] Mimura I, Nangaku M, Kanki Y, Tsutsumi S, Inoue T, Kohro T, et al Dynamic change of chromatin conformation in response to hypoxia enhances the expression of GLUT3 (SLC2A3) by cooperative interaction of hypoxia-inducible factor 1 and KDM3A. Mol Cell Biol. 2012;32(15):3018–32. http://dx.doi.org/10.1128/MCB.06643-11 22645302 10.1128/MCB.06643-11 PMC3434521

[42] Park SJ, Kim JG, Son TG, Yi JM, Kim ND, Yang K, et al The histone demethylase JMJD1A regulates adrenomedullin-mediated cell proliferation in hepatocellular carcinoma under hypoxia. Biochem Biophys Res Commun. 2013;434(4):722–7. http://dx.doi.org/10.1016/j.bbrc.2013.03.091 23583388 10.1016/j.bbrc.2013.03.091

[43] Osawa T, Tsuchida R, Muramatsu M, Shimamura T, Wang F, Suehiro J, et al Inhibition of histone demethylase JMJD1A improves anti-angiogenic therapy and reduces tumor-associated macrophages. Cancer Res. 2013;73(10):3019–28. http://dx.doi.org/10.1158/0008-5472.CAN-12-3231 23492365 10.1158/0008-5472.CAN-12-3231

[44] Niu X, Zhang T, Liao L, Zhou L, Lindner DJ, Zhou M, et al The von Hippel-Lindau tumor suppressor protein regulates gene expression and tumor growth through histone demethylase JARID1C. Oncogene. 2012;31(6):776–86. http://dx.doi.org/10.1038/onc.2011.266 21725364 10.1038/onc.2011.266 PMC4238297

[45] Rondinelli B, Rosano D, Antonini E, Frenquelli M, Montanini L, Huang D, et al Histone demethylase JARID1C inactivation triggers genomic instability in sporadic renal cancer. J Clin Invest. 2015;125(12):4625–37. http://dx.doi.org/10.1172/JCI81040 26551685 10.1172/JCI81040 PMC4665784

[46] Su D, Stamatakis L, Singer EA, Srinivasan R Renal cell carcinoma: Molecular biology and targeted therapy. Curr Opin Oncol. 2014;26(3):321–7. http://dx.doi.org/10.1097/CCO.0000000000000069 24675233 10.1097/CCO.0000000000000069 PMC4232438

[47] Natoli G, Testa G, De Santa F The future therapeutic potential of histone demethylases: A critical analysis. Curr Opin Drug Discov Devel. 2009;12(5):607–15. 19736620

[48] McAllister TE, England KS, Hopkinson RJ, Brennan PE, Kawamura A, Schofield CJ Recent progress in histone demethylase inhibitors. J Med Chem. 2016;59(4):1308–29. http://dx.doi.org/10.1021/acs.jmedchem.5b01758 26710088 10.1021/acs.jmedchem.5b01758

[49] Gale M, Yan Q High-throughput screening to identify inhibitors of lysine demethylases. Epigenomics. 2015;7(1):57–65. http://dx.doi.org/10.2217/epi.14.63 25687466 10.2217/epi.14.63 PMC4356004

[50] Rotili D, Tomassi S, Conte M, Benedetti R, Tortorici M, Ciossani G, et al Pan-histone demethylase inhibitors simultaneously targeting Jumonji C and lysine-specific demethylases display high anticancer activities. J Med Chem. 2014;57(1):42–55. http://dx.doi.org/10.1021/jm4012802 24325601 10.1021/jm4012802

[51] Hashizume R, Andor N, Ihara Y, Lerner R, Gan H, Chen X, et al Pharmacologic inhibition of histone demethylation as a therapy for pediatric brainstem glioma. Nat Med. 2014;20(12):1394–6. http://dx.oi.org/10.1038/nm.3716 25401693 10.1038/nm.3716 PMC4257862

[52] Thinnes CC, England KS, Kawamura A, Chowdhury R, Schofield CJ, Hopkinson RJ Targeting histone lysine demethylases – Progress, challenges, and the future. Biochim Biophys Acta. 2014;1839(12):1416–32. http://dx.doi.org/10.1016/j.bbagrm.2014.05.009 24859458 10.1016/j.bbagrm.2014.05.009 PMC4316176

[53] Mund C, Lyko F Epigenetic cancer therapy: Proof of concept and remaining challenges. Bioessays. 2010;32(11):949–57. http://dx.doi.org/10.1002/bies.201000061 21154865 10.1002/bies.201000061

[54] Guo X, Lu J, Wang Y, Gui Y, Duan X, Cai Z Ascorbate antagonizes nickel ion to regulate JMJD1A expression in kidney cancer cells. Acta Biochim Biophys Sin (Shanghai). 2012;44(4):330–8. http://dx.doi.org/10.1093/abbs/gms004 22318714 10.1093/abbs/gms004

[55] Pereira F, Barbáchano A, Singh PK, Campbell MJ, Muñoz A, Larriba MJ Vitamin D has wide regulatory effects on histone demethylase genes. Cell Cycle. 2012;11(6):1081–9. http://dx.doi.org/10.4161/cc.11.6.19508 22370479 10.4161/cc.11.6.19508

[56] Larkin J, Goh XY, Vetter M, Pickering L, Swanton C Epigenetic regulation in RCC: Opportunities for therapeutic intervention? Nat Rev Urol. 2012;9(3):147–55. http://dx.doi.org/10.1038/nrurol.2011.236 22249190 10.1038/nrurol.2011.236

